# Comparative Radiological Outcomes of Stand-alone Cage versus Cage and Plate in Anterior Cervical Discectomy and Fusion: A Retrospective Cohort Study

**DOI:** 10.1055/s-0044-1801833

**Published:** 2025-01-28

**Authors:** Isam Sami Moghamis, Abduljabbar Alhammoud, Amgad M. Elshoeibi, Abedallah Abudalou, Jawad Derbas, Mutaz Awad Alhardallo, Salahuddeen Abdelsalam, Abdulmoeen Baco

**Affiliations:** 1 Department of Orthopedic Surgery, Hamad General Hospital, Doha, Qatar; 2 Department of Orthopedic Surgery, University of Arizona, Tucson, Arizona; 3 College of Medicine, Qatar University, QU Health, Doha, Qatar

**Keywords:** anterior cervical discectomy and fusion (ACDF), anterior cervical plate, adjacent segment degeneration, stand-alone cage

## Abstract

**Background** Anterior cervical discectomy and fusion (ACDF) is one of the most commonly used techniques for neural decompression in degenerative cervical radiculopathy and cervical myelopathy. Controversies regarding the superiority of cage augmentation with anterior cervical plate remain, yet several surgeons are still performing ACDF with a stand-alone cage (ACDF-SA). Our study aimed to compare the radiological outcomes between the ACDF augmented with anterior cervical plate (ACDF-CPA) and ACDF-SA in single-level cervical degenerative disc disease.

**Methods** A retrospective data review was conducted for patients who underwent ACDF between January 2011 and December 2019. All adult patients who underwent single-level ACDF for cervical radiculopathy and myelopathy with at least 12 months of follow-up were included in the study. Patients who had a systemic infection, trauma injury, history of malignancy, inadequate radiographs, and less than 12 months of follow-up were excluded from the study. Radiological outcomes, including cage subsidence, fusion rate, and adjacent segment degeneration, were assessed by two senior orthopaedic spine fellows. Adjusted risk ratios were used to compare the radiological outcomes of ACDF-SA and ACDF-CPA, adjusting for age and gender.

**Results** A total of 43 patients were included. Among them, 58% of the patients underwent a stand-alone cage ACDF, while 42% had anterior cervical plate augmentation. The overall fusion rate at 6 months was 76%. The ACDF-SA group's fusion rate was 88%, while that of the ACDF-CPA group was 61%. At 12 months, the overall fusion rate was 81% and was comparable between the two groups. Cage subsidence and adjacent segment degeneration rates were similar between the groups at 6 and 12 months. Adjusted relative risk analysis showed a 50% higher probability of fusion at 6 months in the ACDF-SA group compared with the ACDF-CPA group (95% confidence interval [CI]: 1.01–2.22) and a 22% higher probability at 12 months, though not statistically significant (95% CI: 0.90–1.64). Female gender was associated with higher fusion rates and lower subsidence risk at 12 months.

**Conclusion** Augmentation with the anterior cervical plate in ACDF did not show superiority to the conventional stand-alone cage in mono-segmental ACDF. Our study showed similar outcomes regarding cage subsidence, adjacent segment disease, and fusion rates at 12 months. However, the stand-alone cage achieved faster fusion at 6 months than the plate group.

## Introduction

Anterior cervical discectomy and fusion (ACDF) is one of the most commonly used techniques for treating degenerative cervical radiculopathy and cervical myelopathy. 1 The proposed procedure provides both neural decompression at the symptomatic level and segmental stability. Cervical cages have been widely used as a fusion tool in this procedure. They are biocompatible and composed of diverse materials, such as carbon, titanium, and polyetheretherketone, which can be filled with different types of synthetic bone grafts. 1 2

There have been controversies about the superiority of augmentation with anterior cervical plate fixation over stand-alone cage placement. Placing a titanium plate can provide additional stability to the operated segment, preventing the collapse of the interbody fusion device. 3 However, ACDF augmented with anterior cervical plate (ACDF-CPA) alters the normal biomechanical state of the cervical spine, leading to motion obliteration at the fused segment, increasing the stress on the adjacent segment, and increasing abnormal activities, accelerating adjacent segment degeneration. 4 Yet, there is a wide diversity of implant choices. Several surgeons adopt ACDF with a stand-alone cage (ACDF-SA), while others use anterior cervical plate augmentation, aiming for better outcomes and fewer complications. 5 6 7 8 9 10 11 12 13 14

Our study primarily aimed to compare the radiological outcomes between ACDF-SA and ACDF-CPA in single-level cervical degenerative disc disease. The secondary objective was to assess the associations between patient characteristics and radiological outcomes.

## Methods

### Study Design and Ethical Approval

This retrospective cohort study was conducted following approval from the institutional review board of our local medical research center (approval number MRC-01–21–136), with a waiver for informed consent due to the nature of the research.

### Patient Selection

The study included all adult patients (>18 years old) with cervical radiculopathy or myelopathy resulting from single-level cervical degenerative disc disease who had failed conservative treatment and underwent ACDF with either a tantalum stand-alone cage (ACDF-SA) or a cage augmented with an anterior titanium cervical plate (ACDF-CPA) between January 2011 and December 2019. All procedures were performed by attending physicians from the orthopaedic or neurosurgery departments at the same academic institution. The stand-alone cage devices used were nonlocking tantalum cages, while the anterior plate instrumentation consisted of low-profile titanium. The devices were secured to the cervical vertebrae with two cranial screws and two caudal screws. Patients with a history of systemic infection, trauma, malignancy, inadequate radiographs, or less than 12 months of follow-up were excluded from the study.

### Data Collection and Radiological Evaluation

**Fig. 1:**
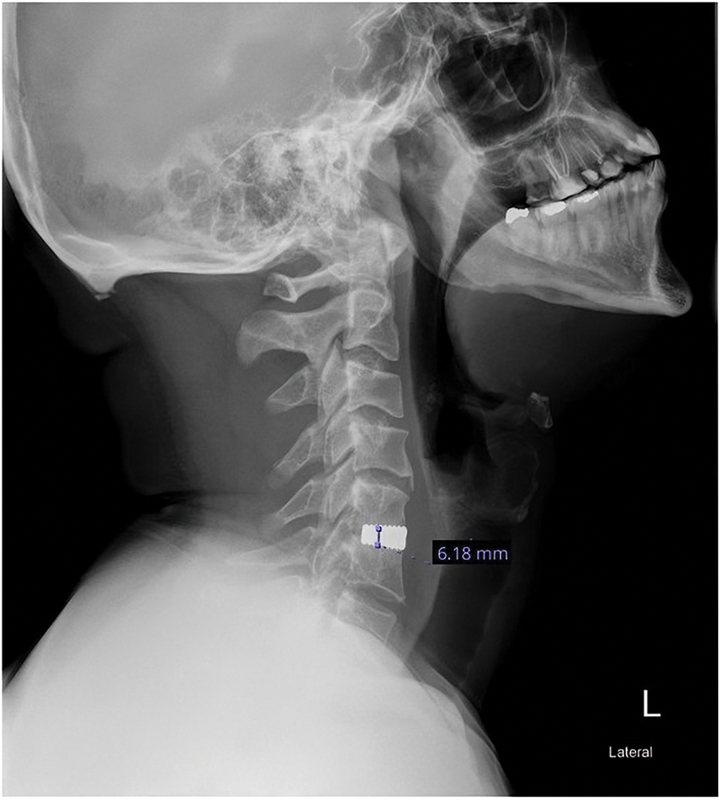
Lateral X-ray of the cervical spine with stand-alone cage, the blue line indicating the midpoint of the lower margin of the upper vertebra and the upper margin of the lower vertebra at the fusion site for which the cage subsidence is measured.

Retrospective data collection was performed using medical records. Patient demographics, including age, gender, comorbidities, operating surgeon (orthopaedics or neurosurgery), and the level of operation, were recorded. Radiological data were reviewed by two senior orthopaedic spine fellows, supervised by a senior spine surgeon. Cervical radiographs were taken immediately postoperatively, at 6 months, and at 12 months. Radiological cage subsidence on lateral cervical spine plain radiographs was defined as a ≥2 mm loss of intervertebral height by comparing postoperative intervertebral heights with those at the last follow-up. The total decrease in intervertebral height was measured between the midpoint of the lower margin of the upper vertebra and the upper margin of the lower vertebra at the fusion site as shown in Fig. 1 .

Intervertebral fusion was assessed using the Oshina criteria, which defines fusion by the presence of bridging trabecular bone between the endplates. Flexion–extension radiographs were evaluated, with less than 1 mm of motion between spinous processes being considered confirmation of successful fusion. 15 Adjacent segment degeneration was assessed 12 months postoperatively using the Hilibrand criteria, including disc space narrowing (>25%), new or enlarged osteophytes, anterior or posterior longitudinal ligament calcification, endplate sclerosis, and magnetic resonance imaging evidence of new disease in the adjacent segment. 16 All radiological outcomes were assessed and measured using the FUJI PACS (Picture Archiving and Communication System) at our institution.

### Statistical Analysis

All statistical analyses were conducted using Stata 17.0 (College Station, Texas, United States). Descriptive statistics were used to summarize demographic and radiological measures. Continuous data were assessed using histograms; normally distributed data were summarized as means and standard deviations, while skewed data were summarized as medians and interquartile ranges. Categorical variables were summarized as numbers and percentages. The chi-square test and Fisher's exact test were used to compare categorical variables. The two-sample *t* -test was used to compare normally distributed continuous data, and the Wilcoxon rank-sum test was applied to skewed data. Adjusted risk ratios (ARRs) were used to compare the radiological outcomes of ACDF-SA and ACDF-CPA, as well as to assess the association between patient characteristics and radiological outcomes. Adjustment was done for age and gender only. The method for estimating ARRs was based on the approach reported by Norton et al. 17

## Results

### Patient Demographics and Operative Characteristics

The demographic and operative characteristics of the included patients are detailed in Table 1 . A total of 43 patients were included in the study, with 58% undergoing surgery with a stand-alone cage, while 42% had the procedure augmented with an anterior cervical plate. The mean age was 49.5 years (standard deviation [SD]: 11.5), with 40% of patients aged between 41 and 50 years. Males comprised 53% of the cohort, and orthopaedic surgeons performed the majority of the surgeries (74%). The most commonly treated spinal level was C5–C6 (49%), followed by C6–C7 (33%), with an average operative time of 147.2 minutes (SD: 38.6). In terms of comorbidities, 21% of the patients were smokers, and 23% had diabetes. Baseline characteristics were generally similar between the two groups, except for diabetes, which was more prevalent in the cage and plate group (39%) compared with the stand-alone cage group (12%).

**Table 1:** Demographic and operative characteristics of included patients

Variable	ACDF-SA	ACDF-CPA	Overall	*p* -Value
*N*	25	18	43	
Age, mean (SD)	47.0 (9.6)	52.9 (13.1)	49.5 (11.5)	0.092
Gender				
Male	12 (48%)	11 (61%)	23 (53%)	0.4
Female	13 (52%)	7 (39%)	20 (47%)	
Operating surgeon				
Orthopaedics	18 (72%)	14 (78%)	32 (74%)	0.74
Neurosurgery	7 (28%)	4 (22%)	11 (26%)	
Operated level				
C3–C4	2 (8%)	3 (17%)	5 (12%)	0.24
C4–C5	1 (4%)	1 (6%)	2 (5%)	
C5–C6	11 (44%)	10 (56%)	21 (49%)	
C6–C7	11 (44%)	3 (17%)	14 (33%)	
C7–T1	0 (0%)	1 (6%)	1 (2%)	
Smoking status	7 (28%)	2 (11%)	9 (21%)	0.26
Cortisone use	1 (4%)	0 (0%)	1 (2%)	1
Diabetes	3 (12%)	7 (39%)	10 (23%)	0.067
Operation time, mean (SD)	143.2 (39.2)	152.8 (38.0)	147.2 (38.6)	0.42

Abbreviations: ACDF-CPA, anterior cervical decompression and fusion-cervical plate augmentation; ACDF-SA, anterior cervical decompression and fusion-stand-alone cage; ASD, adjacent segment disease.

### Fusion Outcomes, Subsidence, and Adjacent Segment Degeneration

As shown in Table 2 , the overall fusion rate at 6 months was 77%, with the stand-alone cage group achieving a higher fusion rate of 88% compared with 61% in the cage and plate group. By 12 months, the overall fusion rate increased to 81%, with similar rates between the stand-alone cage group (88%) and the cage and plate group (72%). Regarding subsidence, 44% of patients experienced cage subsidence at 6 months, rising to 60% by 12 months, with no notable difference between the groups at either time points. Additionally, 74% of patients showed signs of adjacent segment degeneration by 12 months, with 76% in the stand-alone cage group and 72% in the cage and plate group. Importantly, none of the patients required revision surgery during the follow-up period.

**Table 2:** Comparison of radiological and clinical outcomes between ACDF-SA and ACDF-CPA

Factor	Level	ACDF-SA	ACDF-CPA	Overall	*p* -Value
*N*		25	18	43	
Subsidence at 6 months	No	15 (60%)	9 (50%)	24 (56%)	0.51
	Yes	10 (40%)	9 (50%)	19 (44%)	
Subsidence at 12 months	No	10 (40%)	7 (39%)	17 (40%)	0.94
	Yes	15 (60%)	11 (61%)	26 (60%)	
Fusion at 6 months	No	3 (12%)	7 (39%)	10 (23%)	0.067
	Yes	22 (88%)	11 (61%)	33 (77%)	
Fusion 12 months	No	3 (12%)	5 (28%)	8 (19%)	0.25
	Yes	22 (88%)	13 (72%)	35 (81%)	
ASD at 12 months	No	6 (24%)	5 (28%)	11 (26%)	0.78
	Yes	19 (76%)	13 (72%)	32 (74%)	
Subsidence	None	9 (36%)	7 (39%)	16 (37%)	0.66
	6 months	10 (40%)	9 (50%)	19 (44%)	
	12 months	6 (24%)	2 (11%)	8 (19%)	
Fusion	None	3 (12%)	5 (28%)	8 (19%)	0.098
	6 months	22 (88%)	11 (61%)	33 (77%)	
	12 months	0 (0%)	2 (11%)	2 (5%)	
Reoperation	No	25 (100%)	18 (100%)	43 (100%)	

Abbreviations: ACDF-CPA, anterior cervical decompression and fusion-cervical plate augmentation; ACDF-SA, anterior cervical decompression and fusion-stand-alone cage; ASD, adjacent segment disease.

### Comparison of Radiological Outcomes between ACDF-SA and ACDF-CPA

Table 3 compares the adjusted relative risks (RRs) for radiological outcomes between ACDF-SA and ACDF-CPA, with the latter as the reference group. At 6 months, patients in the ACDF-SA group had a 50% higher probability of achieving fusion compared with the ACDF-CPA group, with strong evidence against the model hypothesis at this sample size (RR: 1.50, 95% confidence interval [CI]: 1.01–2.22, *p* = 0.021). By 12 months, there was a 22% increase in probability of fusion in the ACDF-SA group compared with the ACDF-CPA group, however with weak evidence against the model hypothesis (RR: 1.22, 95% CI: 0.90–1.64, *p* = 0.174).

**Table 3:** Association between ACDF-SA and ACDF-CPA in radiological outcomes

Outcome	Adjusted RR	Lower 95% CI	Upper 95% CI	*p* -Value	Reference group
Fusion at 6 months	1.50	1.01	2.22	0.021	ACDF-CPA
Fusion at 12 months	1.22	0.90	1.64	0.174	ACDF-CPA
Subsidence at 6 months	0.59	0.33	1.06	0.071	ACDF-CPA
Subsidence at 12 months	0.90	0.57	1.42	0.660	ACDF-CPA
ASD at 12 months	1.20	0.82	1.76	0.333	ACDF-CPA

Abbreviations: ACDF-CPA, anterior cervical decompression and fusion-cervical plate augmentation; ACDF-SA, anterior cervical decompression and fusion-stand-alone cage; ASD, adjacent segment disease; CI, confidence interval.

For subsidence, there was a 41% reduction in the risk of subsidence at 6 months in the ACDF-SA group compared with ACDF-CPA (RR: 0.59, 95% CI: 0.33–1.06, *p* = 0.071), with some evidence against the null hypothesis. By 12 months, the risk of subsidence was comparable between the two groups, with little difference between the groups (RR: 0.90, 95% CI: 0.57–1.42, *p* = 0.660). Similarly, for adjacent segment disease at 12 months, the ACDF-SA group had a 20% higher risk, but with weak evidence against the model hypothesis at this sample size (RR: 1.20, 95% CI: 0.82–1.76, *p* = 0.333).

### Associations between Patient Characteristics and Radiological Outcomes

Table 4 demonstrates the associations between various exposure variables and radiological outcomes. Female gender was associated with a 52% increased probability of achieving fusion at 6 months (RR: 1.52, 95% CI: 1.05–2.19, *p* = 0.010) and a 45% increased probability at 12 months (RR: 1.45, 95% CI: 1.05–2.01, *p* = 0.009), with strong evidence against the null hypothesis at this sample size. Females also had a 51% lower risk of subsidence at 12 months (RR: 0.49, 95% CI: 0.28–0.86, *p* = 0.002), again with strong evidence against the null hypothesis.

**Table 4:** Association between patient characteristics and radiological outcomes

Exposure variable	Fusion 6 months	Fusion 12 months	Subsidence 6 months	Subsidence 12 months	ASD 12 months
Gender					
Male	**Reference**				
Female	1.52 (1.05–2.19), 0.010 a	1.45 (1.05–2.01), 0.009 a	0.91 (0.48–1.73), 0.782	0.49 (0.28–0.86), 0.002 a	0.87 (0.60–1.26), 0.461
Age group, years					
30–40	**Reference**				
41–50	0.93 (0.54–1.60), 0.800	1.14 (0.68–1.89), 0.612	0.79 (0.42–1.49), 0.477	0.94 (0.62–1.43), 0.780	1.23 (0.67–2.29), 0.482
51–60	1.22 (0.75–1.98), 0.404	1.25 (0.76–2.04), 0.351	0.52 (0.20–1.36), 0.146	0.73 (0.40–1.34), 0.294	1.54 (0.85–2.81), 0.104
> 60	1.25 (0.76–2.07), 0.352	1.28 (0.77–2.13), 0.302	–	0.32 (0.10–1.09), 0.008 a	1.38 (0.68–2.82), 0.367
Smoking	1.02 (0.69–1.51), 0.939	1.11 (0.84–1.47), 0.470	1.26 (0.59–2.69), 0.573	1.05 (0.53–2.08), 0.891	0.73 (0.37–1.43), 0.300
Diabetes	1.05 (0.74–1.50), 0.769	0.96 (0.68–1.36), 0.822	1.52 (0.85–2.71), 0.183	1.33 (0.85–2.06), 0.231	0.97 (0.62–1.54), 0.912
Operated level					
C3–C4	**Reference**				
C4–C5	0.67 (0.15–2.99), 0.556	0.78 (0.23–2.62), 0.674	–	–	0.41 (0.07–2.56), 0.263
C5–C6	1.24 (0.60–2.58), 0.529	1.24 (0.62–2.48), 0.510	0.70 (0.21–2.36), 0.611	1.59 (0.40–6.27), 0.417	1.21 (0.51–2.87), 0.638
C6–C7	1.16 (0.53–2.51), 0.700	1.34 (0.67–2.67), 0.352	0.97 (0.29–3.20), 0.961	1.84 (0.43–7.85), 0.312	1.44 (0.61–3.41), 0.336

Abbreviations: ASD, adjacent segment disease.

Note: Cells displayed as RR (95% CI), *p* -value.

a Statistically significant *p* -values.

Age categories did not show strong associations with fusion or subsidence, except for patients over 60, who had a 68% reduced risk of subsidence at 12 months (RR: 0.32, 95% CI: 0.10–1.09, *p* = 0.008), with strong evidence against the null hypothesis. Smoking and diabetes were not associated with any of the radiological outcomes, with weak evidence observed across all variables. Regarding the operated level, no clear associations were found with fusion or subsidence, though the C5–C6 level showed a 59% increase in the risk of subsidence at 12 months (RR: 1.59, 95% CI: 0.40–6.27, *p* = 0.417), but this result had weak supporting evidence.

## Discussion

Disc height following ACDF typically increases in the immediate postoperative period but gradually returns to preoperative levels, or slightly above or below them. 1 18 Studies report variable rates of cage subsidence with stand-alone cages, ranging from 8 to 32%, typically occurring within the first 3 months after surgery without further progression. 2 18 19 20 21 22 In contrast, anterior cervical plate augmentation has been associated with a reduction in cage subsidence rate. 2 21 23 However, recent meta-analyses have shown no significant difference in subsidence rates between the two groups, indicating that the stand-alone cage does not increase the risk of cage subsidence in mono-segmental ACDF, even in long-term outcomes. 24 25 26 Our findings were consistent with this, showing no significant association between type of fixation and cage subsidence at 12-month follow-up.

In our study, the fusion rates at 12 months were similar across the two groups. However, signs of fusion appeared earlier in ACDF-SA when compared with the ACDF-CPA group with a higher probability of fusion at 6 months. This may be related to the continued micro-motions at the fusion site with ACDF-SA, which are minimized by anterior cervical plate augmentation. Moreover, the application of a plate requires a greater disruption to the soft tissues, microvasculature, and periosteal layer. Overall, our results align with previous literature, which reports satisfactory arthrodesis rate regardless of plating status. 4 27 28 Furthermore, Zhu et al found similar fusion rates in multilevel ACDF in both groups at 3-year follow-up. 29

Adjacent segment degeneration is a common complication following ACDF, affecting up to 47% of the patients. 30 31 Biomechanical studies suggest that the use of titanium plates increases stress on adjacent disc spaces, potentially accelerating adjacent segment degeneration. 32 33 Zhou et al reported a higher postoperative risk of adjacent segment disease in patients with ACDF-CPA compared with ACDF-SA, and Zhang et al found similar results in mono-segmental ACDF. 25 However, in our study, there was no significant difference in the incidence of adjacent segment degeneration between the two groups at 12-month follow-up.

## Limitations

There are some limitations in our study. First, the retrospective design limited the range of variables that could be assessed. Although CT scans are ideal for providing more detailed information on fusion and other outcomes, we relied on X-rays due to the constraints of the study's retrospective nature. The small sample size resulted in wide CIs, affecting the precision of our estimates and limiting the robustness of our conclusions. Additionally, the procedures were performed by different surgeons, potentially contributing to variability in surgical techniques and outcomes. Lastly, the short-term follow-up may not fully capture long-term complications or outcomes, particularly for conditions like adjacent segment disease or late-onset subsidence.

## Conclusion

Augmentation with the anterior cervical plate in ACDF did not show superiority to the conventional stand-alone cage in mono-segmental ACDF at 12 months. Our study showed similar outcomes regarding cage subsidence, adjacent segment disease, and fusion rates at 12 months. However, the stand-alone cage achieved faster fusion at 6 months compared with the plate group. Future studies are needed to compare the results of ACDF-SA and ACDF-CPA groups prospectively.
